# Seroprevalence and risk factors of canine distemper virus in the pet and stray dogs in Haa, western Bhutan

**DOI:** 10.1186/s12917-020-02355-x

**Published:** 2020-05-13

**Authors:** Tshering Dorji, Tenzin Tenzin, Kuenga Tenzin, Dawa Tshering, Karma Rinzin, Waraphon Phimpraphai, Michel de Garine-Wichatitsky

**Affiliations:** 1 Kasetsart University, Faculty of Veterinary Medicine, Bangkok, Thailand; 2 District Veterinary Hospital, Department of Livestock, Gasa, Bhutan; 3 National Centre for Animal Health, Department of Livestock, Thimphu, Bhutan; 4 District Veterinary Hospital, Department of Livestock, Haa, Bhutan; 5 Animal Health Division, Department of Livestock, Thimphu, Bhutan; 6 CIRAD, UMR ASTRE, Montpellier, France; 7 ASTRE, Univ Montpellier, CIRAD, INRA, Bangkok, Thailand

**Keywords:** Free-roaming dog, Canine distemper virus, Protected area, Western Bhutan

## Abstract

**Background:**

Canine Distemper Virus (CDV) is a highly contagious virus belonging to family *Paramyxovirade*, genus *Morbillivirus* and responsible for high morbidity and mortality in dogs worldwide. Infected domestic dogs can cause spillover infections to wild carnivores that are in contact. We conducted a seroprevalence survey of CDV in domestic dogs in two areas of western Bhutan (Haa district) located at the periphery of the Jigme Khesar Strict Nature Reserve, which is home to several endangered wildlife. A total of 238 serum samples, 119 each from the pet and stray dog, were collected during summer and winter seasons. Samples were tested for CDV antibodies using a sandwich enzyme-linked immune-sorbent assay (ELISA) test.

**Results:**

The seroprevalence of CDV was found to be 11.3% (95% CI 6.7–14.2). Dogs sampled during winter were less likely to test seropositive against CDV antibodies than those sampled during summer (adjusted odds ratio: -2.6; 95% CI: − 1.2–6.1). Dogs in good body condition were found to be more likely to test seropositive against CDV than dogs in poor condition and obese dogs (adjusted odds ratio: 2.2; 95% CI: 0.1–5.9). There were no significant differences in the seroprevalence of CDV among different sexes, breeds and age classes, pet and stray dogs and between the two study sites.

**Conclusions:**

Our study indicates that CDV seroprevalence was equally distributed among pet and stray dogs. We suggest strengthening the management practices of dogs through responsible dog ownership, dog population management and waste management to minimize the transmission risk of infectious diseases to wildlife.

## Background

Canine Distemper Virus (CDV) belongs to a group of negative single-stranded and non-segmented RNA virus of species *Canine Morbillivirus,* genus *Morbillivirus*, family *Paramyxovirade* [1]. CDV is antigenically and genetically closely related to the other members of the *morbillivirus* genus, which includes measles virus, rinderpest virus and, peste des petits ruminant virus [2]. CDV is a highly contagious virus that can cause fatality in the domestic dog [3, 4]. The distemper virus affects a wide range of animal families, both domestic and wild, including *Canidae* (dog, fox, wolf, raccoon dog), *Mustelidae* (ferret, mink, skunk, wolverine, martin, badger, otter), *Procyonidae* (raccoon, coati), *Ailuridae* (red panda, giant panda), *Ursidae* (bear), *Elephantidae* (Asian elephant), some primates (Japanese monkey), *Viverridae* (civet), *Pinnipedia* (seals, walrus, sea lion), *Felidae* (large cats), *Myrmecophagidae (Tamandua tetradactyla*) commonly known as Southern tamandua, and most recently reported in giant anteater (*Myrmecophaga tridactyla*) in Brazil [5–10].

Bhutan is considered as major biological hotspots due to the exceptional diversity and originality of its flora and fauna [11, 12]. Several vulnerable and endangered species are present in Bhutan, such as the common leopard (*Panthera pardus)*, clouded leopard (*Neofelis nebulosa*), Snow Leopard (*Panthera uncia)* and Red Panda (*Ailurus fulgens*) [13–16]. These animals, may be directly threatened by infectious diseases such as CDV [5, 17–19]. The national conservation policies allow farmers to live within and adjacent to the protected areas in Bhutan, under specific rules regulating the use of natural resources and agriculture practices which presents both challenges and opportunities to conservation programs [20].

In Bhutan, Canine Distemper disease (CD) is common and there have been recurrent outbreaks in dogs resulting in mass mortality [21, 22]. Vaccination against CDV is done only in a limited number of pet dogs (individually owned) while the stray dogs are not vaccinated and thus remain susceptible to infection. A previous study in the Thimphu capital city indicated high seroprevalence (49.7%) of CDV among dogs, but most positive cases were probably due to vaccination since the majority of pet dogs in Thimphu are vaccinated against the disease [23]. To our knowledge, no other studies have been conducted to determine CDV prevalence in dogs in other parts of Bhutan. The spillover infection of CDV from dogs to wild canids and felids is a potential threat for carnivore conservation [3, 17, 18]. Therefore, understanding the prevalence of CDV in free-roaming domestic dogs potentially in contact with susceptible wild carnivores in the periphery of the protected area is important to inform policies regarding dog population management and wildlife conservation. In this paper, we investigated the seroprevalence and the risk factors associated with CDV among pet and stray dogs in the Haa area, western Bhutan, located in the buffer zone of Jigme Khesar Strict Nature Reserve.

## Results

### Dog demographic characteristics

A total of 238 dogs were sampled, of which 51% were female in Esue rural *geog* and 49% in Katsho semi-urban *geog* (sub-districts). Most of the dogs were local/non-descriptive breeds in Esue (90%) and Katsho *geog* (69%). The mean age of the dogs sampled from both areas was 3.25 year, with a minimum of 7 months and a maximum of 12 years. The distribution of dog age classes was similar between Esue and Katsho, although the age class [13–24 month] was overrepresented in Katsho and older dogs (> 49) were overrepresented in Esue (t = 2.8546, df = 27, *P* < 0.01). The detailed characteristics of the sampled dogs are shown in Table 1.

**Table 1:** Demographic characteristics of dogs sampled in Esue rural *geog* (*n* = 119) and Katsho semi-urban area (n = 119)

Variables	Esue/rural n (%)	Katsho/semi-urban n (%)	χ^**2**^ test significance
**Sex**			
Female	61 (51)	58 (49)	0.79
Male	58 (49)	61 (51)	
**Breed**^a^			
Pure/cross	12 (10)	37 (31)	0.0001
Local	107 (90)	82 (69)	
**Dog category/classification**			
Owned	62 (52)	71 (60)	
Stray	57 (48)	48 (40)	0.29
**Age (months)**			
4–12	10 (8.4)	8 (6.7)	0.008
13–24	11 (9.2)	19 (16)	
25–48	93 (78.2)	90 (75.7)	
> 49	5 (4.2)	2 (1.6)	
**Physical body condition score**			
5-Obese	10 (8)	18 (15)	
4-Fat	72 (60)	57 (48)	
3-Normal	8 (7)	12 (10)	0.28
2-Thin	15 (13)	17 (14)	
1-Emaciated	14 (12)	15 (13)	
**Neuter status**			
Yes	58 (49)	52 (44)	0.51
No	61 (51)	67 (56)	

Note: ^a^Dog breed: Local means non-descript and mongrel dog; pure/cross mean pure breed (e.g. German Shepherd) and crosses between local and one identified pure breed

### Factors associated with seroprevalence of CDV in dogs

Twenty-seven of the 238 dogs (11.3, 95% CI: 6.7–14.2) were found to be seropositive to CDV. There was no significant difference in the prevalence of CDV between males and females, between pure/cross and local breeds, pet, and stray dogs, among different age categories, between neutered and entire dogs, and between the two study sites (Esue and Katsho). Only two variables, the season of sampling and body condition scores, were associated with CDV seropositivity. Dogs sampled during winter were less likely to test seropositive against CDV antibodies compared to those sampled during summer (adjusted odds ratio: -2.6; 95% CI: − 1.2-6.1). Dogs with a body condition score of 5 (Obese), were more likely to test positive against CDV than other physical body condition (adjusted odds ratio: 2.2;95% CI: 0.1–5.9) (Table 2).

**Table 2:** Final multivariable model of factors associated with seroprevalence of canine distemper virus in dogs sampled in Haa, Bhutan

Variable	Coefficient	SE	***p***-value	Odds Ratio (95% CI)
Constant				–
	−1.60944	0.77460	0.037	
**Season**				
Summer	–	–	–	1.00
Winter	−19.76853	1533.91099	0.989	−2.6 (−1.2–6.1)
**Physical body condition score**				
Emaciated	–	–	–	1.00
Thin	−0.095	1.092	0.930	0.8 (−1.6–2.9)
Average	0.182	1.329	0.89	1.2 (0.2–4.7)
Fat	0.255	0.824	0.757	2.2 (0.1–5.9)
Obese	2.303		0.028	1.9 (0.2–6.6)

## Discussion

Our study estimated the prevalence of CDV in both pet and stray dogs in rural and semi-urban communities of Haa district in Western Bhutan. The ELISA test used in this study only detected IgG antibodies. Thus, we could not differentiate between recent and past infections, and between vaccination and natural infections [24, 25]. However, since owners and livestock officials confirmed that the sampled dogs were not vaccinated against CD, we believe that the positive cases detected during our study are due to natural infections with CDV. Vaccination of dogs against CD remains expensive in most developing countries and it is logistically challenging to vaccinate stray and free-roaming dogs [26–28]. However, it is important to note that since the ELISA test is not 100% sensitive and specific, some samples could have been tested false positive or false negative and thus, this result should be interpreted cautiously.

**Fig. 1:**
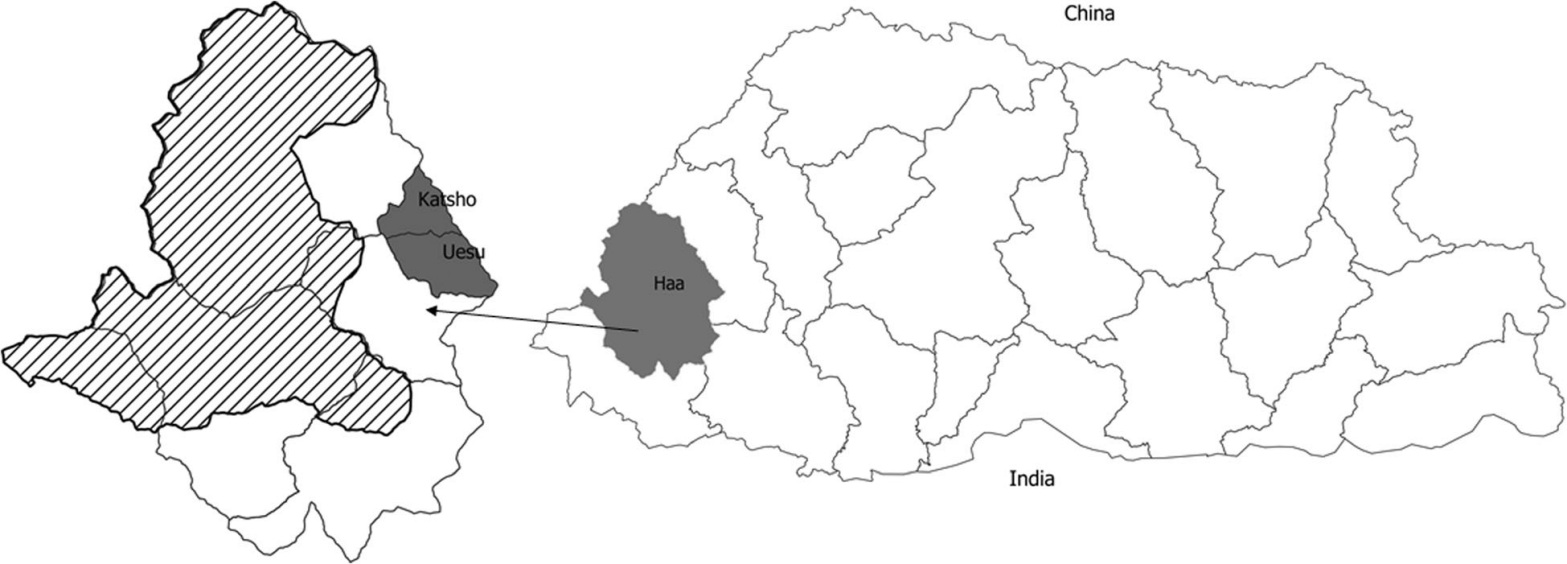
Map of Bhutan showing the study area (Katsho and Esue *geogs* in Haa district) and the Jigme Khesar Strict Nature Reserve area (line shaded area). This map was prepared using QGIS Development Team (2019), QGIS Geographic Information System, Open Source Geospatial Foundation Project (http://qgis.osgeo.org) and was not taken from another source

The prevalence of CDV natural infection in high-density domestic dog populations in our study area [29] emphasizes the risk of transmission among the domestic dog population, and of spillover infection to wild carnivores populations. In a previous study [30], we demonstrated that interactions between pet dogs and wildlife in an adjacent protected area of the study site were possible (Fig. 1). This is a concern for wildlife conservation as it poses significant transmission risks to several endangered wild canid and felid species, as observed in studies conducted elsewhere [9, 31, 32]. Farmers from the study site area reported observing frequent interactions between domestic dogs and wildlife, mostly at the periphery of agriculture land, and there is evidence of free-roaming dogs attacking Himalayan black bear in the study area [30].

Like any other infectious disease, the maintenance of CDV in a host population requires that the size of the susceptible population remains above the threshold. As wild carnivores occur at low densities, it is expected that the circulation of CDV is not maintained independently [33, 34]. For instance, in Bhutan, the density of the tiger is estimated at 0.24 tigers per 100km^2^ whereas the density of the snow leopard is estimated at 1.08 snow leopard per 100km^2^ [35, 36]. However, despite their low densities; these two endangered species face significant risks of infection with CDV from domestic free-roaming dogs due to large stray dog populations at the periphery of protected areas, and occasionally inside, often resulting in dog-wildlife conflicts [37–39]. Additionally, the large density of free-roaming dogs in the country is considered as one of the major threats to Red panda conservation [40].

The current study findings of higher seropositivity to CDV during the summer season compared to winter may be associated with the probability of survival of CDV during warmer climatic conditions, as demonstrated in a similar study conducted elsewhere [41]. It has been documented that a sustained dog population management and pet ownership are the only solutions to reduce dog population, which would, in turn, reduce dog-wildlife interactions and resulting pathogen transmission [18, 34]. The sustained dog population management, through sterilization and waste management [23, 28, 30], responsible pet ownership by providing proper food, shelters and health care services are some of the strategies that can reduce dog population in the country [42, 43]. The government of Bhutan has placed high priority towards dog population and waste management and approved a special flagship program in the current plan. This is expected to have positive impacts by reducing the free-roaming dog population, improving animal welfare, reducing disease transmission (including CDV), thereby preventing spillover infection into wildlife [44–46]. In addition, vaccination of domestic free-roaming dogs against CDV may reduce disease transmission and benefit wildlife conservation as observed elsewhere [26, 41, 47].

## Conclusions

Our serological study demonstrated that CDV circulates in domestic dog populations in the periphery of protected areas that hosts a range of endangered wildlife species potentially threatened by CDV. Since CDV is a highly infectious disease that can spill over to wildlife, particularly wild canids, we recommend that dog population control and management through responsible dog ownership, animal birth control program and waste management be carried out in areas where domestic dogs are likely to share habitat with wildlife. In addition, vaccination of both pet and stray dogs against CDV, within and outside protected areas where dog-wildlife interfaces occur, should be considered.

## Methods

### Study area

The study was conducted at the periphery of the Jigme Khesar Strict Nature Reserve (JKSNR), in Haa Dzongkhag (district), the western part of Bhutan (Fig. 1). This protected area was created in 1993 by the Royal Government of Bhutan. This is the only protected area without permanent human settlements, except for few migratory yak herds. A total of 29 species of mammals, 161 species of birds, 64 species of butterfly and seven species of fish have been recorded within this park boundary [48]. JKSNR is home to endangered species such as common leopard (*Panthera pardus)*, Red Panda (*Ailurus fulgens*), Clouded leopard (*Neofelis nebulosa*), Snow Leopard (*Panthera uncia)*, and Tiger (*Panthera tigris)* [35, 36, 49, 50], which are all listed as classified as vulnerable (VU) or endangered (EN) in the ICUN Red list of threaten species [14]. These species are vulnerable to CDV spill-over infection from domestic free-roaming dogs, as has been experienced elsewhere [9, 18, 19]. This reserve area is also part of the transboundary conservation landscape - the Kangchenjunga landscape – that extends up to Sikkim in India and Nepal. There are six *geogs* (sub-districts) under Haa Dzongkhag, and we selected one semi-urban *geog* (Katsho) and one adjacent rural *geog* (Esue), both located 6 km away from JKSNR boundary. The people in these *geogs* keep domestic animals and yaks that graze within the nature reserve area. Katsho *geog* is located at an altitude ranging from 2850 to 3100 m above sea level and has an area of 42.8 square meters. It is the smallest of the six *geogs* in Haa district which caters to 250 households and 1385 people. Esue *geog* has an area of 66.5 km^2^ and is located at an altitude of 2521 to 4076 masl. There are 11 villages with 255 households with a total population of 1907 [51].

### Sampling and laboratory analysis

Using a priori CDV prevalence of 50% [23] and with a 10% error and 95% confidence interval, a total of 238 serum samples were required to be collected from the two study sites. We collected two batches of serum samples: one batch in summer (August to September 2018) and one during winter (December 2018–February 2019). The samples were collected opportunistically during the Catch-Neuter-Vaccinate-Release campaigns (CNVR) of dogs conducted by the district livestock services of Haa during the summer season and through door-to-door visits for pet dogs during winter. Although there was no history of dog’s vaccination against CDV in the study areas, only dogs that were above 4 months of age were included in the sampling to minimize the possible confounding influence of maternally derived antibodies. The age of pet dogs was collected through owner interview, whereas for stray dogs it was assessed by examining the dentition when under anesthesia for surgical operation during CNVR program. A minimum of 2 ml of blood was collected from the cephalic vein and the serum samples were obtained following centrifugation. The samples were shipped to the National Centre for Animal Health laboratory in Thimphu and preserved at -20 °C until analysis. The samples were tested using a canine reactive ELISA kit as per the manufacturer instructions [24]. The other characteristics of the dogs such as sex, breed, body condition score and neutering status were collected at the time of sampling.

### Data analysis

Data were entered, cleaned and managed in Microsoft Excel (Microsoft Excel, Redmon, USA) and were analyzed in R statistical software (version 3.5) using the packages “dplyr”,“descr”, “forcats”, “lmtest”, and “LogisticDx” [52]. The descriptive analysis was conducted by calculating the percentages and frequencies of the variables. The factors associated with seropositivity of the samples were assessed by performing logistic regression analysis. The associated factors investigated were age, sex, breed (local vs pure/cross) physical body condition score (obese, healthy, fair, weak and emaciated), sterilization status of the dog (yes vs no), category of dog (pet vs stray), the season of sample collection (summer vs winter) and sites of sampling (rural villages vs semi-urban). Age of the dogs (continuous) were re-categorized into four groups for regression analysis. Initially, a univariable logistic regression was performed with the sero-positive status of the dogs (1 or 0) as an outcome and the above-mentioned factors as the predictor variables. The variables with a *p*-value of < 0.25 were selected for the multivariable logistic regression model and those variables with *p* < 0.05 were considered significant.

## Data Availability

All data generated or analysed during this study are included in the result section. The full datasets will be made available from the corresponding author on request.

## Abbreviations

CD: Canine distemper
CDV: Canine distemper virus
CNVR: Catch neuter vaccinate release
ELISA: Enzyme-linked immunosorbent assay
IUCN: International Union for Conservation of Nature
